# Screening for depression: The added value of actigraphy and smartphone-based intensive sampling of depressive affect and behaviors

**DOI:** 10.1192/j.eurpsy.2021.314

**Published:** 2021-08-13

**Authors:** O. Minaeva, H. Riese, F. Lamers, N. Antypa, M. Wichers, S. Booij

**Affiliations:** 1 Department Of Psychiatry, University of Groningen, University Medical Center Groningen, Groningen, Netherlands; 2 Department Of Psychiatry, Vrije Universiteit, Amsterdam UMC, Amsterdam Public Health research institute, Amsterdam, Netherlands; 3 Department Of Clinical Psychology, Leiden University, Institute of Psychology, Leiden, Netherlands

**Keywords:** Prediction model, Experience Sampling Method, Actigraphy, Depression

## Abstract

**Introduction:**

In many countries, depressed individuals often first visit primary care settings for consultation, but a considerable number of clinically depressed patients remains unidentified. Introducing additional screening tools may facilitate the diagnostic process.

**Objectives:**

This study aims to examine whether Experience Sampling Method (ESM)-based measures of depressive affect and behaviors can discriminate depressed from non-depressed individuals. In addition, the added value of actigraphy-based measures was examined.

**Methods:**

We used data from two samples to develop and validate prediction models. The development dataset included 14 days of ESM and continuous actigraphy of currently depressed (n=43) and non-depressed individuals (n=82). The validation dataset included 30 days of ESM and continuous actigraphy of currently depressed (n=27) and non-depressed individuals (n=27). Backward stepwise logistic regression analyses were applied to build the prediction models. The performance of the models was assessed with the goodness of fit indices, calibration curves, and discriminative ability (AUC, the area under the receiver operating characteristic curve).

**Results:**

In the development dataset, the discriminative ability was good for the actigraphy model (AUC=0.790) and excellent for the ESM (AUC=0.991) and combined-domains model (AUC=0.993). In the validation dataset, the discriminative ability was reasonable for the actigraphy model (AUC=0.648) and excellent for the ESM (AUC=0.891) and combined-domains model (AUC=0.892).

**Conclusions:**

ESM is a good diagnostic predictor and is easy to calculate, and, therefore, holds promise for implementation in clinical practice. Actigraphy shows no added value to ESM as a diagnostic predictor, but might still be useful when active monitoring with ESM is not feasible.

**Disclosure:**

No significant relationships.

